# Bn*PME* is progressively induced after microspore reprogramming to embryogenesis, correlating with pectin de-esterification and cell differentiation in *Brassica napus*

**DOI:** 10.1186/s12870-016-0863-8

**Published:** 2016-08-11

**Authors:** María-Teresa Solís, Eduardo Berenguer, María C. Risueño, Pilar S. Testillano

**Affiliations:** Pollen Biotechnology of Crop Plants group, Biological Research Center, CIB-CSIC, Ramiro de Maeztu 9, 28040 Madrid, Spain

**Keywords:** Pectin methylesterase, Pectin esterification, Cell wall remodeling, Cell reprogramming, Microspore embryogenesis, Pollen, Gametophytic development, Totipotency, Differentiation, Proliferation

## Abstract

**Background:**

Pectins are one of the main components of plant cell walls. They are secreted to the wall as highly methylesterified forms that can be de-esterified by pectin methylesterases (PMEs). The degree of methylesterification of pectins changes during development, PMEs are involved in the cell wall remodeling that occurs during diverse plant developmental processes. Nevertheless, the functional meaning of pectin-related wall remodeling in different cell types and processes remains unclear. In vivo, the microspore follows the gametophytic pathway and differentiates to form the pollen grain. In vitro, the microspore can be reprogrammed by stress treatments becoming a totipotent cell that starts to proliferate and follows the embryogenic pathway, a process known as microspore embryogenesis.

**Results:**

To investigate if the change of developmental programme of the microspore towards embryogenesis involves changes in pectin esterification levels, which would cause the cell wall remodeling during the process, in the present study, dynamics of PME expression and degrees of pectin esterification have been analysed during microspore embryogenesis and compared with the gametophytic development, in *Brassica napus*. A multidisciplinary approach has been adopted including Bn*PME* gene expression analysis by quantitative RT-PCR, fluorescence in situ hybridization, immuno-dot-blot and immunofluorescence with JIM5 and JIM7 antibodies to reveal low and highly-methylesterified pectins. The results showed that cell differentiation at advanced developmental stages involved induction of Bn*PME* expression and pectin de-esterification, processes that were also detected in zygotic embryos, providing additional evidence that microspore embryogenesis mimics zygotic embryogenesis. By contrast, early microspore embryogenesis, totipotency and proliferation were associated with low expression of Bn*PME* and high levels of esterified pectins.

**Conclusions:**

The results show that the change of developmental programme of the microspore involves changes in pectin esterification associated with proliferation and differentiation events, which may cause the cell wall remodeling during the process. The findings indicate pectin-related modifications in the cell wall during microspore embryogenesis, providing new insights into the role of pectin esterification and cell wall configuration in microspore totipotency, embryogenesis induction and progression.

## Background

The cell wall, as the most external layer of the plant cell, is involved in a number of functions such as interaction with the environment, growth and development, intercellular communication, and the determination of cell size and shape. Plant cell walls are highly complex structures composed of diverse polysaccharides (cellulose, hemicellulose and pectins), enzymes and structural proteins which change their composition during plant growth and adaptation [1]. Pectins are major components of the primary plant cell walls; they are secreted into the cell wall in a highly methylesterified form and can be de-esterified *in muro* by pectin methylesterases or PMEs [2]. The methylesterification of pectins affects to their homogalacturonan domain (HGA) and changes significantly during plant growth and development [3]. PMEs are involved in important physiological processes such as microsporogenesis, pollen growth, seed germination, root development, polarity of leaf growth, stem elongation, fruit ripening, and loss of tissue integrity [4–14].

Microspore embryogenesis is a widely used method to generate genetic variability by obtaining microspore-derived embryos and double-haploid plants with many applications for plant breeding [15]. This process involves the reprogramming of the immature pollen— the microspore— towards a different developmental pathway and the onset of proliferation and differentiation events, which finally lead to embryo formation and haploid and double-haploid plant regeneration [16, 17]. Changes in various cell activities and in the structural organization of subcellular compartments have been reported to accompany the microspore reprogramming process in some herbaceous and woody species [16, 18–23]. Different studies have indicated that somatic embryogenesis is accompanied by modifications in the structure and molecular composition of cell walls [24]. Moreover, many of the molecular markers of somatic embryogenesis and organogenesis have been found in cell walls [25–28]. Specifically, studies in *Capsicum annuum* have reported differences in the distribution pattern of the major cell wall polymers, xyloglucan and the rhamnogalacturonan II pectin domain, as well as the proportion of esterified and non-esterified pectins in gametophytic and embryogenic development [27, 29]. An unusually thick cell wall under the exine was reported in embryogenic microspores and proembryos at early stages of microspore embryogenesis in several other species [16, 26, 30]. Although some plant cell wall polymers are regulated during plant development, the functional meaning of wall changes in different cell types and processes remains unclear.

Pectin methylesterases (PMEs, EC 3.1.1.11) catalyze the specific removal of methyl esters from the linear homogalacturonan (HGA) backbone of pectins within plant cell walls [3, 31]. The de-methylesterified HGA can either form Ca2+ bonds or become a target for pectin-degrading enzymes, such as polygalacturonases, affecting the texture and rigidity of the cell wall [2, 32]. PMEs are ubiquitous enzymes [2] that have been identified in all plant tissues and organs such as fruits, leaves, flowers, stems and roots [33–35] and in some of plant cell wall-degrading microorganisms or insects [36]. Nevertheless, experimental data on the action of PMEs *in planta* are limited and many aspects of their functions and regulation remain to be elucidated. In various plant species such as banana, cork oak, olive and pepper, recent data have provided evidence of changes in pectins during somatic embryogenesis [35, 37] and microspore embryogenesis [26, 27, 30, 37]; however, there are no data on the PME during pollen development and microspore embryogenesis. Data generated by sequencing projects has shown gene families of several homogalacturonan-modifying enzymes, including pectin methylesterases, in several plant species (reviewed in [38]). However, the information available on PME genes is scarce on *Brassicas*. In *Brassica campestris*, only a few sequences of putative PME isoforms have been annotated (NBCI accession numbers KF268317, KF268318), without any functional or expression information available to date. In *Brassica olearacea*, a novel PMEI (PME inhibitor) gene has been characterized recently [39]. In *Brassica napus*, to our knowledge, only one Bn*PME* gene sequence has been identified and characterized [40], and it is this gene sequence that has been used for expression analyses in the present work.

JIM5 and JIM7 monoclonal antibodies have been reported as particularly useful in revealing the patterns of pectin esterification [41]. JIM5 binds preferably to at least four contiguous non-esterified GalA residues and labels the relatively non-esterified pectin epitopes. By contrast, JIM7 binds to methylesterified residues with adjacent or flanking non-esterified GalA residues and therefore shows the presence of relatively highly methylesterified pectin epitopes [42]. Several studies on antigen distribution detected by the antibodies JIM5 and JIM7 in different plant tissues and organs have provided evidence that the de-esterification of pectins is involved in specific developmental processes, specifically the changes in the ratio of esterified to non-esterified pectins, and differential distribution of the two forms in cell walls [43–45].

To investigate if the change of developmental programme of the microspore towards embryogenesis involves changes in pectin esterification levels accomplished by PMEs, which would suggest the remodeling of the cell wall during the process, we have analysed the dynamics of Bn*PME* gene expression and degrees of pectin esterification during two developmental programmes— the gametophytic development and the stress-induced microspore embryogenesis—in *Brassica napus* using a multidisciplinary approach. Expression patterns of Bn*PME* were studied by qRT-PCR and fluorescence in situ hybridization (FISH). In vivo localization of PME was assessed by GFP-tagged PME in transformed tobacco leaves. JIM5 and JIM7 antibodies recognizing pectins with a low and high degree of methylesterification, respectively, were used for both immuno-dot-blot and immunofluorescence assays, which were analysed by confocal microscopy, during specific stages of the two developmental pathways. The results showed an increase in Bn*PME* expression with embryogenesis progression, and the developmental regulation of the methylesterification status of pectins during microspore embryogenesis and gametophytic development.

## Methods

### Plant material

*Brassica napus* L. cv. Topas donor plants were grown from seeds generated in the Biological Research Center (CSIC, Madrid) greenhouse [17]. Donor plants were grown under controlled conditions at 15 °C day, 16 h photoperiod, and 10 °C night in a growth chamber. Vacuolated microspores and differentiated pollen grains were isolated from anthers at the corresponding developmental stages. Microspore embryogenesis was induced in isolated microspore cultures by a 32 °C treatment, as described [17]. Microspores for in vitro culture were selected from donor plants at the developmental stage of vacuolated microspore, the most responsive stage for embryogenesis induction [17, 46].

### Fixation and cryoprocessing for microscopic analysis

Freshly isolated microspores, pollen grains and in vitro samples from different culture times were collected and fixed overnight at 4 °C with 4 % paraformaldehyde in phosphate-buffered saline (PBS). After fixation, samples were processed by three main methods to obtain: semithin resin sections, semithin cryosections and cryostat sections.

For structural analysis and immunofluorescence, fixed samples were dehydrated and embedded in Historesin resin at 4 °C. Semithin sections were collected on slides; some of them were stained with toluidine blue, for structural analysis, or iodide cytochemistry, for preferential staining of starch [26], and observed under bright field microscopy. Other resin sections were used for immunofluorescence.

For immunofluorescence and FISH assays, fixed samples were processed by two main protocols. Isolated microspores, pollen grains and small samples from the first culture stages were cryoprotected in 2.3 M sucrose, cryofixed in liquid propane and cryosectioned in a cryoultramicrotome (Ultracut E Reichert equipped with FC4 cryounit), whereas fixed large culture samples of the advanced embryogenesis stages and zygotic embryos excised from seeds were embedded in OTC, frozen on dry-ice, and sectioned in a cryostat (Leica Cm 1800). Finally, specimens were mounted on slides coated with APTES (3-aminopropyltriethoxysilane, Sigma), stored at 4 °C until use for immunofluorescence and FISH.

### Antibodies

The antibodies used in this study were JIM7 and JIM5 rat monoclonal antibodies (PlantProbes, Leeds, UK) that recognized highly and low-methylesterified pectins respectively [41]. JIM7 binds to methylesterified residues of homogalacturonan with adjacent or flanking non-esterified GalA residues but does not bind to non-esterified homogalacturonan and therefore shows the presence of relatively highly methylesterified pectin epitopes. JIM5 monoclonal antibody binds preferably to at least four contiguous non-esterified GalA residues, therefore it recognizes partially methyl-esterified epitopes of homogalacturonan and non-esterified homogalacturonan.

### Immunofluorescence

Cryostat sections (20–40 μm thickness) were permeabilized by dehydration-rehydration in methanol series and treatment with 2 % cellulose (Ozonuka R-10) for 1 h at room temperature, washed in PBS and incubated in 5 % bovine serum albumin (BSA) in PBS for 10 min. In contrast, semithin sections (2-3 μm thickness) were directly washed with PBS and blocked with 5 % BSA. Then, both types of sections were treated equally for immunofluorescence as previously described [47]. Sections were incubated with the corresponding primary undiluted antibodies and after washing in PBS three times (5 min each), the signal was revealed with Alexa Fluor 488-labelled anti-rat antibodies (Molecular Probes, Cat. no. A-11001) diluted 1:25 in PBS for 45 min in the dark. Finally, the slides were counterstained with 1 mg/ml DAPI (4’, 6-diamidino-2-phenylindole; Fluka) solution for 10 min and analyzed in a confocal microscope (Leica TCS-SP5). Negative controls were obtained replacing the primary antibody by PBS.

### Immuno-dot-blot assay

The assay was performed essentially as previously described with minor modifications [29]. Freshly isolated microspores, pollen grains and in vitro samples from different culture times were used. Extracts were obtained from 25 mg samples collected at different time points of gametophytic and embryogenic development; they were homogenized in liquid nitrogen and afterwards in 50 ml of buffer solution containing 50 mM Tris–HCl pH 7.2, 50 mM 1,2-cyclohexylenedinitrilotetraacetic acid (CDTA), and 25 mM dithiothreitol and centrifuged at 7000 rpm for 10 min. at 4 °C. The concentration of the resulting supernatants was determined and all samples were adjusted to a concentration of 1 mg/ml. For immuno-dot-blot assays with JIM5 and JIM7 antibodies, 5 μl aliquots of adjusted supernatants were applied to nitrocellulose membrane (Millipore; Bedford, MA) and stained for total protein detection with Ponceau Red, as loading control. Finally, the epitopes recognized by the antibodies were revealed by treatment with a nitroblue tetrazolium, bromo-chloroindolyl-phosphate (NBT-BCIP) mixture.

### Reverse-transcription PCR

Freshly isolated vacuolated microspores and pollen grains from anthers, and in vitro embryogenesis samples from cultures at different time-points and corresponding to several developmental stages (proembryos, globular and cotyledonary embryos) were collected. Total RNA from samples was isolated with the RNeasy® Plant Minikit (Qiagen) according to the manufacturer’s instructions. One microgram of total RNA was used for the RT reaction using the Superscript™ II reverse transcriptase enzyme (Invitrogen). The oligonucleotides used for Bn*PME* expression analysis were: 5′ GGAGCGTCGTTGATGGATGG 3′ and 3′ GTAACCTCGTTCGCCTGACC 5′ (accession AY036606) from the sequence of the *Brassica napus* pectin methylesterase-like gene [40]. cDNA was amplified by PCR using the HotMaster™ Taq DNA polymerase (Eppendorf). PCR products were detected on 1 % agarose gels stained with ethidium bromide. Band intensity was expressed as relative absorbance units. Each band density was first normalized by dividing it by the density of the actin II band in the same lane (to compensate for the variations in the cDNA loading onto the gel). The relative increase or decrease in gene expression at specific developmental stages were calculated by dividing the normalized band density from each stage by that from the vacuolated microspore, which was selected as reference. Consequently, the relative density of the vacuolated microspore band is presented as 1.

### Quantitative real-time PCR (qRT-PCR)

Quantitative gene expression analysis was performed by qRT-PCR with in vitro culture samples at selected developmental stages of microspore embryogenesis: vacuolated microspore (starting point to induce the process), proembryos (early embryogenesis stage) and globular embryos (advanced embryogenesis stage, when embryo differentiation initiates). The expression analysis of the pectinmethylesterase gene (*BnPME*) was performed by quantitative real-time PCR using the SsoAdvanced™ Universal SYBR®Green Supermix on the iQ™5 Real-Time PCR Detection System (Biorad). As PCR templates, cDNA was generated from total RNA isolated from the different culture samples at the analyzed stages, using the Superscript™ II reverse transcriptase enzyme (Invitrogen), according to [48].

The oligonucleotides used were: 5′ GGAGCGTCGTTGATGGATGG 3′ and 3′ GTAACCTCGTTCGCCTGACC 5′ from the sequence of the *PME* gene of *Brassica napus* (AY036606) which encodes a pectin methylesterase-like gene [40]. All qPCR reactions were run in triplicates. Thermocycle settings were as follows: initial denaturation of 30 s at 95 °C, followed by forty cycles, each consisting of 5 s at 95 °C, 30 s at 56 °C. After each run, a dissociation curve was acquired to check for amplification specificity by heating the samples from 65 to 95 °C. Serial dilutions of cDNA were used to determine the efficiency curve of each primer pair according to [49]. Actin II (*ACT*) was used as internal reference gene. At the end of the PCR cycles, the data was analyzed with the Bio-Rad CFX Manager 3.0 (3.0.1224.1015) (Biorad), using the Livak calculation method [50].

### Fluorescence *in situ* hybridization (FISH)

Total cDNA was obtained from total RNA of samples, as described before, by RT reaction using the Superscript™ II reverse transcriptase enzyme (Invitrogen). Amplification of cDNA from the *Brassica napus PME*-*like* sequence, complete cds, was performed by PCR amplification with the same Bn*PME* primers mentioned above for the RT-PCR assays. Amplified fragments of around 500 bp were isolated from an agarose gel and cloned using a pGEM® T-Easy cloning system (Promega). dig-RNA-PME probes were generated by in vitro transcription using the DIG-RNA-labeling kit (Roche).

Cryostat sections were permeabilized by dehydration-rehydration in methanol series and treatment with 2 % cellulose (Ozonuka R-10) for 1 h, washed and dried. Semithin cryosections were washed in PBS to remove the sucrose and dried.

RNA/RNA in situ hybridization was performed as described [48] using dig-PME RNA probes diluted 1/50 in hybridization buffer at 50 °C, overnight. Post-hybridization washes were performed in 4xSSC, 2xSSC and 0,1xSSC. Hybridization signal was detected by immunofluorescence with anti-digoxigenin antibodies, as previously described [48]. After washing in PBS, sections were counterstained with DAPI, mounted in Mowiol, and observed in a confocal microscope (Leica TCS-SP5). Controls were performed with the sense probe.

### In vivo subcellular localization of PME

The PME-GFP fusion protein was generated using the Gateway™ cloning technology developed by Invitrogen (Carlsbad, CA, USA). Primers were synthesized by Roche Applied Biosystems. The construct was expressed under the control of the 35S promoter. Four-week-old *Nicotiana tabacum* (cv. Petit Havana) plants grown at 25 °C were used for *Agrobacterium tumefaciens* (strain GV3101) mediated stable DNA integration [51]. The bacterial optical density (OD 600) used for plant transformations was 0.05. Lower epidermis of transformed leaves was analyzed between 48 to 72 h after infection. Appropriate controls were done in parallel with wild-type plants and infiltrated tobacco leaves with a GFP construct without PME. Image analysis was carried out with a confocal laser scanning microscope (Leica TCS-SP5) under the Ar laser excitation line of 488 nm to detect the fluorescence signals of GFP and by excitation at 633 nm to detect the autofluorescence of chlorophyll.

## Results

### Bn*PME* expression pattern in the two microspore developmental pathways, gametophytic and embryogenic, and in zygotic embryos

**Fig. 1:**
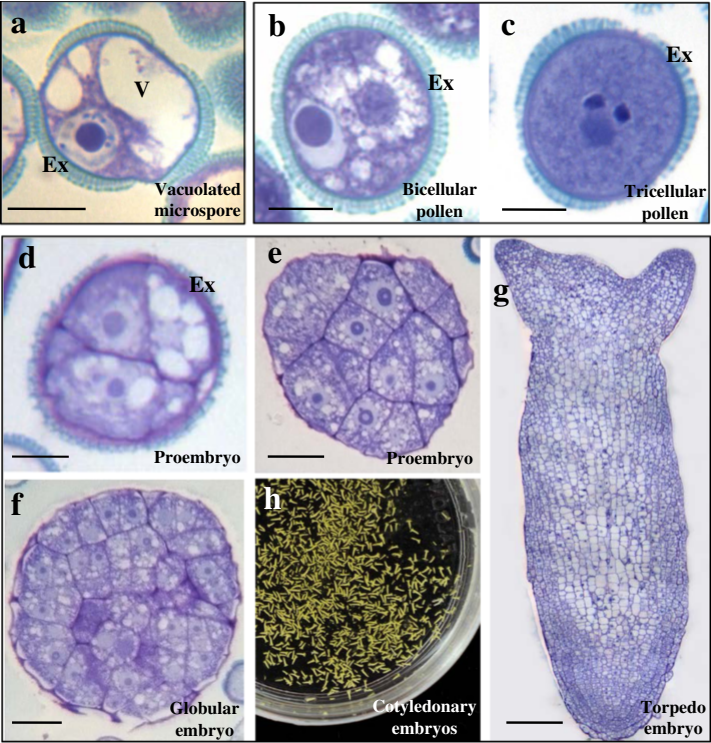
Main stages of the two microspore pathways: gametophytic development and microspore embryogenesis. **a**-**g** Semithin sections, toluidine blue staining. **a** Vacuolated microspore. **b**-**c** Gametophytic development, in vivo. **b** Bicellular pollen. **c** Tricellular mature pollen. **d**-**h** Microspore embryogenesis, in vitro. **d** Two-cell proembryo. **e** Multicellular proembryo. **f** Globular embryo. **g** Late torpedo embryo. **h** Panoramic view of cotyledonary embryos in the Petri dish. Ex: exine, V: vacuole. Bars: **a**-**d**, 10 μm; **e**, **f**, 20 μm; g, 50 μm

The vacuolated microspore is the responsive developmental stage for induction of embryogenesis [46, 48]. It was characterized by a large cytoplasmic vacuole which pushed the nucleus towards a peripheral location (Fig. 1a). At this stage, the microspore exhibited an inner thin wall, the intine, which was surrounded by the outer sporopollenin pollen wall, the exine. When the gametophytic development was followed, the vacuolated microspore underwent an asymmetric division leading to the formation of the bicellular pollen grain containing the small generative cell inside the cytoplasm of the larger vegetative cell (Fig. 1b). Later, the generative cell divided forming the sperm cells, giving rise to the tricellular pollen (Fig. 1c). However, after the application of a heat treatment (32 °C) to induce embryogenesis in vitro, the vacuolated microspore divided symmetrically forming two-cell proembryos whose cells and nuclei were of similar size and organization (Fig. 1d). After subsequent divisions, multicellular proembryos were formed (Fig. 1e). At later stages, after the exine had broken down and cell proliferation increased, globular embryos were formed (Fig. 1f). As embryogenesis progressed, embryos elongated giving rise to heart-shaped and torpedo embryos (Fig. 1g), which finally increased in size and differentiated leading to the formation of mature cotyledonary embryos (Fig. 1h).

**Fig. 2:**
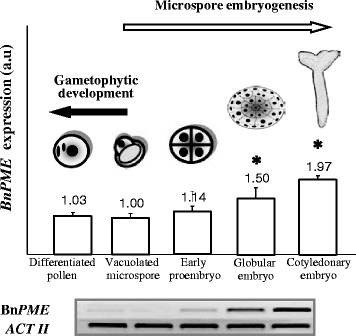
Expression analysis of Bn*PME* gene by RT-PCR during gametophytic development and microspore embryogenesis. Semiquantitative RT-PCR representative agarose electrophoresis gels of the amplification products. Histogram expresses relative changes in the *PME* bands intensities at different developmental stages: vacuolated microspore, differentiated pollen (gametophytic development), early proembryo, globular embryo, cotyledonary embryo (microspore embryogenesis). Columns represent means of mRNA expression of Bn*PME* ± SE (bars) of three independent experiments with three replicates each, in arbitrary units (a.u.). Asterisks indicate significant differences with the vacuolated microspore (Student’s *t*-test at *P* ≤ 0.05)

The temporal expression pattern of *Brassica napus* pectin methylesterase (Bn*PME*), was firstly analysed by RT-PCR at different stages of pollen development and microspore embryogenesis; Bn*PME* codifies for one of the enzymes responsible for de-methylesterification of pectin HGA within cell walls. The stages selected for RT-PCR analysis were “vacuolated microspore”, as the starting phase for the two developmental pathways, “differentiated pollen” for the gametophytic development, and “early proembryo”, “globular embryo” and “cotyledonary embryo” for the microspore embryogenesis pathway. RT-PCR analysis revealed that Bn*PME* expression was developmentally up-regulated at advanced stages of microspore embryogenesis whereas its expression was low after microspore reprogramming and embryogenesis initiation, in early proembryos, as well as during pollen development (Fig. 2). The expression value of the vacuolated microspore was considered as the unit for comparison with the other stages. Bn*PME* expression was progressively induced during microspore embryogenesis progression; globular and torpedo embryos showed higher expression levels than vacuolated microspores, as did cotyledonary embryos, which exhibited the highest expression level (Fig. 2).

**Fig. 3:**
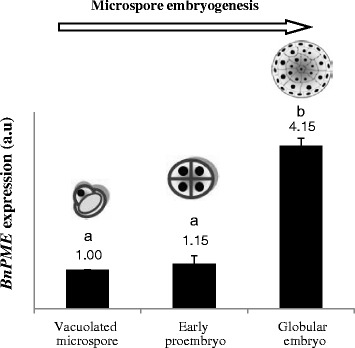
Expression analysis of Bn*PME* gene by quantitative RT-PCR during stress-induced microspore embryogenesis. Histogram expresses relative changes of expression at different developmental stages: vacuolated microspore (starting point, before reprogramming induction), early proembryo, and globular embryo. Each column represents the mean of two independent reactions with three replicates of each reaction. Transcript levels were normalized to vacuolated microspore levels. Bars indicate the SEM. Different letters on columns indicate significant differences at *P* < 0.05

For a more accurate and quantitative analysis of the changes in gene expression during microspore embryogenesis, qRT-PCR assays were performed in selected key developmental stages: “vacuolated microspore” (starting point to induce the process), “early proembryos” (early embryogenesis stage) and “globular embryos” (advanced embryogenesis stage in which embryo differentiation initiates). Quantitative PCR results supported the data obtained by semiquantitative RT-PCR and showed no significant differences in Bn*PME* expression between vacuolated microspores, before reprogramming, and early proembryos (Fig. 3). By contrast, the gene was highly up-regulated at the advanced embryogenesis stage of globular embryos; expression of globular embryos increased 4-fold in comparison with expression of vacuolated microspores (Fig. 3).

**Fig. 4:**
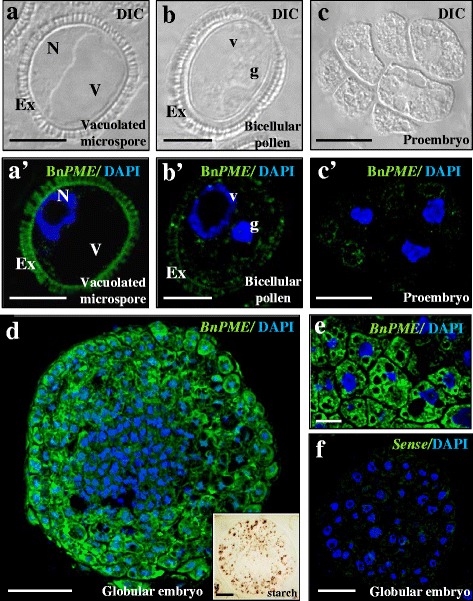
*In situ* Bn*PME* expression by FISH during gametophytic development and microspore embryogenesis. **a**, **b**, **c** are the same structures as in **a**’, **b**’, **c**’. **a**, **b**, **c** DIC images (Differential Interference Contrast, Nomarsky). **a**’, **b**’, **c**’, **d**, **e**, **f** Confocal merged images of fluorescence provided by DAPI staining of nuclei (in blue) and fluorescence in situ Bn*PME* hybridization signal (in green). **a**, **a**’ Vacuolated microspore. **b**, **b**’ Gametophytic development, in vivo, bicellular pollen. **c**, **c**’, **d**, **e**, **f** Microspore embryogenesis, in vitro. **c**-**c**’ Proembryo. **d** Globular embryo. (Inset in d) Globular embryo stained with I_2_IK for starch. **e** Detail of globular embryo peripheral cells at higher magnification. **f** FISH control with the sense probe in globular embryo. Ex: exine, N: nucleus, V: vacuole, g: generative nucleus, v: vegetative nucleus. Bars: **a**, **a**’, **b**, **b**’, **c**, **c**, 10 μm; **d**, **e**, **f**, 20 μm

To analyse the spatial expression pattern and the subcellular distribution of Bn*PME* transcripts, fluorescence in situ hybridization (FISH) was carried out, followed by confocal microscopy analysis. In the gametophytic pathway, the FISH experiments results showed very low hybridization signal in cytoplasms at the stages of vacuolated microspore (Fig. 4a, a’), bicellular pollen (Fig. 4b, b’) and tricellular pollen. In the embryogenic pathway, after embryogenesis induction, early proembryos also showed low hybridization signal in the cytoplasm (Fig. 4c, c’). The exine exhibited unspecific autofluorescence in some samples (Fig. 4a’, b’). At later developmental stages of the embryogenic pathway, the FISH signal was progressively higher, especially in the cells of the outer layers of advanced globular embryos (Fig. 4d). Hybridization signal was specifically found in the cytoplasm, where transcripts were localized, whereas no signal was found in nuclei, vacuoles or cell walls (Fig. 4d, e). Globular embryos exhibited two regions with different cell types: an inner region showing cells with dense cytoplasms, large nuclei and small vacuoles, which is typical organization of proliferating cells, similar to early proembryo cells, and an outer area containing large cells with big vacuoles and starch deposits, as revealed by iodide specific staining (inset in Fig. 4d), which is typical cytoplasmic organization of cells in differentiation. In torpedo embryos, a differential Bn*PME* expression pattern was also found; differentiating cells of peripheral regions presented a higher hybridization signal than proliferating cells of inner embryo regions. Control FISH experiments performed with sense probes did not show fluorescence in any cellular region of any samples (Fig. 4f).

**Fig. 5:**
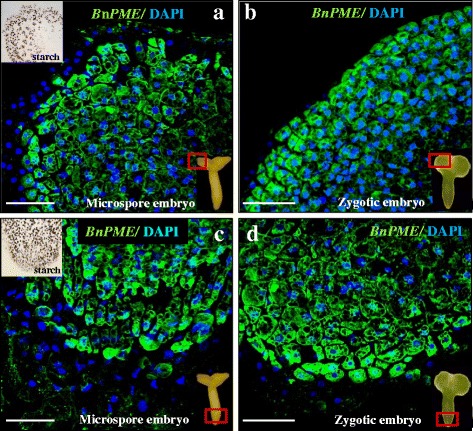
*In situ* *PME* expression by FISH in cotyledonary microspore embryos and zygotic embryos. Confocal merged images of fluorescence provided by DAPI staining of nuclei (in blue) and fluorescent in situ hybridization signal (in green). **a**, **c** Microspore embryo. **b**, **d** Zygotic embryo. **a**, **b** Cotyledon region of embryos, as indicated by the square. **c**, **d** Radicular region of embryos, as indicated by the square. Insets in **a** and **c**: Iodide specific staining for starch, analogous cotyledonar and radicular embryo regions as in “**a**” and “**c**”. Bars: 50 μm

*In situ *expression analysis was also carried out in cotyledonary microspore embryos and zygotic embryos obtained from seeds. FISH results showed that the patterns of Bn*PME* expression were similar in the two types of embryos (Fig. 5). High fluorescence hybridization signal appeared in embryos from both origins, microspore (Fig. 5a, c) and zygote (Fig. 5b, d), specifically in the cytoplasm of differentiating cells of the cotyledonary and radicular regions, characterized by starch accumulations as revealed by iodide specific staining (insets in Fig. 5a, c), Some embryo cells at inner regions of cotyledons and radicular tip showed lower fluorescence hybridization signal and no starch deposits (Fig. 5a, b, c, d and insets). Cells located in the outer layers of cotyledons and in the root cap of embryos did not show FISH signal (Fig. 5).

### PME: GFP in vivo subcellular localization

**Fig. 6:**
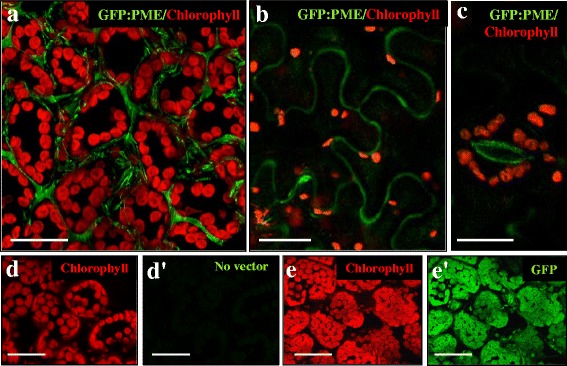
In vivo GFP: PME subcellular localization in transformed tobacco leaves. Confocal images of fluorescence provided by chlorophyll autofluorescence (in red) and GFP fluorescence (in green). **a**, **b**, **c** GFP: PME transformed leaves, merged images of red and green channels of mesophyll **a**, pavement epidermal **b** and stomatal guard **e** cells. **d**, **d**’ Wild type leaf. **e**, **e**’ GFP transformed leaf. **d**, **e** Red channel. **d**’, **e**’ Green channel. Bars: 25 μm

To analyse the subcellular localization of the Bn*PME* protein in vivo, its gene was expressed in a heterologous system. PME gene was translationally fused to the GFP and this construct was cloned under the control of the constitutive 35S promoter and used in transient expression experiments in transformed *Nicotiana tabacum* leaves. Confocal in vivo analysis of the transformed tobacco leaves with the PME-GFP construct showed an intense green fluorescence signal in the cell walls of mesophyll cells (Fig. 6a), indicating that, after translation and processing, PME was directed towards the cell wall where pectin modification would occur *in muro*. No PME: GFP signal was found inside mesophyll cells, with only their numerous chloroplasts exhibiting an intense red signal corresponding to autofluorescence of chlorophyll (Fig. 6a). PME: GFP signal was also found in the thin cell walls of the pavement epidermal cells, outlining the typical shape of these cells (Fig. 6b). PME: GFP was also localized in the walls of guard cells that surround the stomata in the epidermis (Fig. 6c), showing an intense fluorescence signal, especially in the inner walls facing the pore side (these wall regions are usually thicker than the rest of guard cell wall). By contrast, wild type tobacco leaves, used as negative control of the experiment, did not show any green fluorescence signal (Fig. 6d, d’). On the contrary, the infiltration with the GFP construct without PME provided unspecific green fluorescence signal located all over the mesophyll cells, even inside the chloroplasts (Fig. 6e, e’), supporting the specificity of the signal of PME: GFP and the localization in cell walls of the PME protein encoded by the Bn*PME* gene analysed.

### Temporal patterns of pectin esterification in the two microspore developmental pathways, gametophytic and embryogenic

**Fig. 7:**
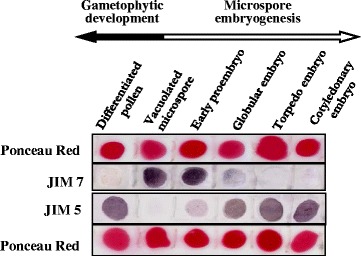
Temporal patterns of esterified and non-esterified pectins during gametophytic development and microspore embryogenesis. Immuno-dot-blot assays at different developmental stages: vacuolated microspore, differentiated pollen (gametophytic development), early proembryo, globular, torpedo and cotyledonary embryo (microspore embryogenesis). Ponceau Red staining for total proteins and immuno-dot blot of the same strip is shown for each antibody

Immuno-dot-blot assays were carried out with the antibodies JIM7 and JIM5 recognizing highly and low methyl-esterified pectins respectively, to determine the differences in the presence of the two forms of pectins during the two developmental programmes. The dot-blot analysis was performed at the stages previously described, “vacuolated microspore”, as starting phase for the two pathways, “differentiated pollen” for the gametophytic development, and “early proembryos”, “globular embryos”, “torpedo embryos” and “cotyledonary embryos” for microspore embryogenesis. Equal sample amounts were used in all developmental stages as revealed by Ponceau Red staining of the membranes (Fig. 7). The results showed different distribution patterns of esterified and non-esterified pectins in the two developmental pathways (Fig. 7). JIM7 immuno-dot-blot signal showed high levels of esterified pectins in vacuolated microspores and early proembryos, whereas it diminished at later stages of microspore embryogenesis. During gametophytic development, JIM7 signal decreased, and was particularly low in mature pollen. On the contrary, JIM5 signal, corresponding to non-esterified pectins, was low in vacuolated microspore and progressively increased with pollen differentiation, as well as with microspore embryogenesis progression (Fig. 7). JIM5 signal reached its maximum levels in cotyledonary embryos and differentiated pollen (Fig. 7).

Regarding the ratio between esterified and non-esterified pectins in each developmental stage, vacuolated microspores and early proembryos exhibited very similar dot blot patterns, high JIM7 and low JIM5 signals (Fig. 7). Globular embryos displayed a significant increase in non-esterified pectins (JIM5) accompanied by a decrease in the levels of esterified pectins (JIM7) (Fig. 7). In subsequent embryogenic stages, the difference between non-esterified and esterified pectins became greater, JIM5 signal increased markedly while JIM7 signal decreased significantly in late embryo stages (Fig. 7).

### *In situ * localization of esterified and non-esterified pectins in the two microspore developmental pathways, gametophytic and embryogenic

Esterified and non-esterified pectin distribution patterns were analysed by immunofluorescence with JIM7 and JIM5 antibodies at different stages of the two developmental pathways. Confocal microscopy analysis of JIM5 and JIM7 immunofluorescence assays revealed specific changes in the distribution pattern of esterified and non-esterified pectins in the cell wall during the developmental processes analysed.

**Fig. 8:**
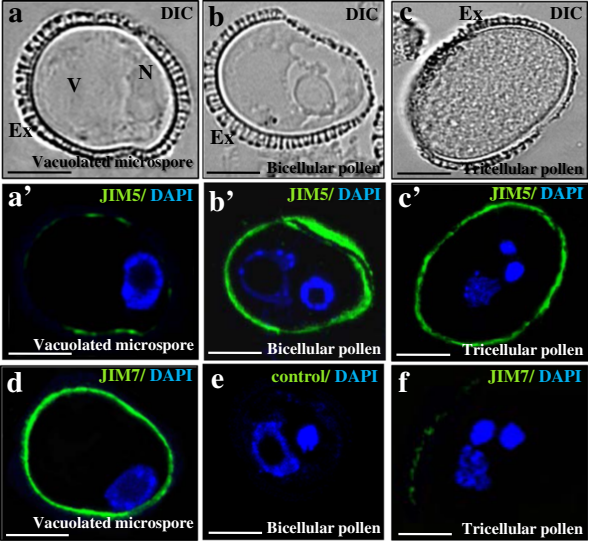
Immunolocalization of esterified and non-esterified pectins during gametophytic development. **a**, **b**, **c** are the same structures, visualized by DIC (Differential Interference Contrast, Nomarsky) as in **a**’, **b**’, **c**’. **a**’, **b**’, **c**’ Immunofluorescence of esterified (JIM7 antibody) and non-esterified (JIM5 antibody) pectins during gametophytic development. **a**, **a**’, **d** Vacuolated microspore; **b**, **b**’, **e** Bicellular pollen; **c**, **c**’, **f** Tricellular mature pollen. **a**’, **b**’, **c**’ Merged images of fluorescence provided by DAPI staining of nuclei (in blue) and JIM5 immunofluorescence signal (in green). **d**, **f** Merged images of fluorescence provided by DAPI staining of nuclei (in blue) and JIM7 immunofluorescence signal (in green). **e** Control immunofluorescence experiment avoiding the first antibody. Ex: exine, N: nucleus, V: vacuole. Bars: 10 μm

In vacuolated microspores (Fig. 8a, a’, d), the immunofluorescence assays provided an intense JIM7 signal over the intine (the inner wall under the exine) (Fig. 8d), indicating a high content of esterified pectins. However, no signal was detected with JIM5 antibody in vacuolated microspores (Fig. 8a’). In the bicellular and tricellular pollen stages of the gametophytic pathway, the distribution patterns for JIM7 and JIM5 signals were opposite to those of vacuolated microspores (Fig. 8b, b’, c, c’, f). An intense signal of non-esterified pectins, revealed by JIM5, was found on the intine, (Fig. 8b’, c’), while the immunofluorescence signal with JIM7 was very low (Fig. 8f), in both the bicellular and tricellular pollen stages. In all cases, the fluorescent signal was localized on the intine layer, without any fluorescent signal on the exine or any other cell compartment (Fig. 8). Control immunofluorescence experiments avoiding the first antibody did not provide signal, at any developmental stage (Fig. 8e).

**Fig. 9:**
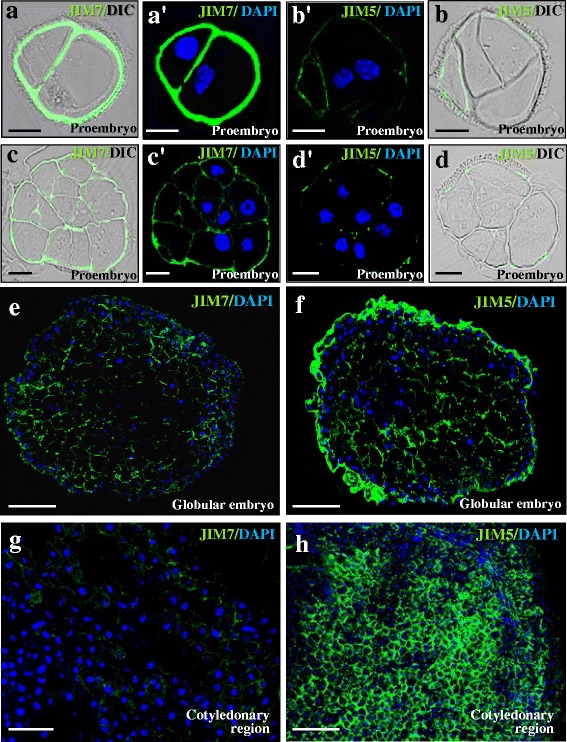
Immunolocalization of esterified and non-esterified pectins during microspore embryogenesis. Confocal merged images of immunofluorescence signal (green) and DAPI staining of nuclei (blue). For some stages, DIC image (Differential Interference Contrast, Nomarsky) of the same section is shown to reveal the structure (left side for each pair of images). **a**, **a**’, **c**, **c**’, **e**, **g** JIM7 immunofluorescence. **b**, **b**’, **d**, **d**’, **f**, **h**) JIM5 immunofluorescence. **a**-**d** Two-cell and early proembryos. **e**, **f** Globular embryos. **g**, **h** Cotyledonary embryos. Bars: **a**-**d**) 10 μm; **e**-**h**) 25 μm

After the induction of microspore embryogenesis, early proembryos containing two or a few cells displayed thin walls which showed a high fluorescence signal with JIM7 antibody (Fig. 9a, a’, c, c’); on the contrary, very low signal (or no signal at all) was observed in early proembryo cell walls with JIM5 antibody (Fig. 9b, b’, d, d’). In small proembryos, still surrounded by the exine, the intine also showed an intense JIM7 signal (Fig. 9a, a’). At later developmental stages, in globular and torpedo embryos, a stronger JIM5 signal was detected in the walls of the outer differentiating cells (Fig. 9f); by contrast, a weak signal (or none at all) was observed in the walls of the inner proliferating cells (Fig. 9f). Labeling with JIM7 antibody was lower in cell walls of advanced globular and torpedo embryos (Fig. 8e). In cotyledonary embryos, the differences between immunofluorescence signals of the two antibodies became greater. JIM5 labeling was intense in cell walls, especially in differentiating regions like cotyledon tips (Fig. 9h), while JIM7 signal was very low in cells of cotyledons (Fig. 9g). In controls carried out without the primary antibodies no signal was detected in any developmental stage. These patterns of distribution clearly correlated with the results of the immuno-dot-blot assays reported above.

## Discussion

### Microspore embryogenesis progression involves induction of Bn*PME* expression and increasing pectin de-esterification levels in cell walls

In this study, we have investigated if the change of developmental programme of the microspore towards embryogenesis involved changes in pectin esterification levels, accomplished by PME activity, which would suggest cell wall remodeling during the process. The expression analysis of Bn*PME*, performed by different and complementary methodologies — qRT-PCR and fluorescence *in situ *hybridization (FISH) — showed that Bn*PME* is induced during microspore embryogenesis progression, specifically in embryo differentiating cells characterized by starch accumulations. This Bn*PME* expression pattern fully correlated with the increase of non-esterified pectins in cell walls revealed by JIM5 antibody.

Interestingly, similar *in situ* Bn*PME* expression patterns to those of microspore embryos were also found in zygotic embryos. A small number of previous studies have reported structural and gene expression similarities between embryos from the two origins, microspore and zygote, in *Capsicum annuum*, *Quercus suber* and *Brassica napus* [16, 27, 48, 52]. The present PME results provide additional evidence that microspore embryogenesis mimics zygotic embryogenesis.

The methylesterification status of cell wall homogalacturonans (HGA), mediated through the action of PMEs, influences the biophysical properties of plant cell walls that are important parameters for cell elongation and growth in differentiation events [7, 43]. Differential temporal and spatial expression of *PME* genes has been proposed as a major mechanism to regulate the endogenous PME activity [34]. Gradients of decreasing HGA methylesterification levels and increasing PME activities have been found from growing/proliferating to maturing/differentiating tissues [43, 53, 54]. Also in embryogenesis, several PMEs are expressed during silique development in *Arabidopsis* [55], some of them specifically in the embryo, endosperm or seed coat [56, 57]. The high Bn*PME* expression that we observed at later stages of microspore-derived and zygotic embryos could lead to a higher PME activity and pectin de-esterification, changing cell wall properties for embryo differentiation. It has been suggested that the activity of the PME *HIGHLY METHYL ESTERIFIED SEEDS* (*HMS*) promotes cell expansion during embryogenesis, and the mutant *hms1* resulted in altered embryo morphology [57]. Our results suggest that after microspore reprogramming towards embryogenesis, the PME-mediated configuration of pectins could be a crucial factor that may contribute to the temporal regulation of biomechanical properties of cell walls through the balance between highly and low-esterified pectins.

Recent studies have revealed that auxin, among other factors, contributes to the cell wall remodeling during organogenesis and growth, a process in which de-esterification of pectins is required [58, 59]. The progression of plant embryogenesis is associated with polar auxin accumulation [60] and recent reports have indicated that increasing auxin biosynthesis, action and polar transport are required for microspore embryogenesis initiation and progression in *Brassica napus* [61]. Further work would be necessary to investigate if auxin is involved in cell wall remodeling by increasing PME expression, during microspore embryogenesis.

### PME protein is localized in cell walls

To elucidate the in situ subcellular localization of PME protein encoded by the Bn*PME* gene analysed in this study, expression of the *BnPME*: *GFP* fusion construct was analysed in transformed *Nicotiana tabacum* leaves. The in vivo analysis of the PME: GFP construct under confocal laser microscopy showed that PME protein is exclusively localized in the cell walls of both mesophyll and epidermal cells of tobacco leaves, as well as in cell walls of the stomatal guard cells. First attempts to localize PME using immunofluorescence microscopy indicated that, at the tissue level, PMEs were mainly located in the epidermis and in the cell junctions of the cortical parenchyma, suggesting PME as an early marker of differentiation [62]. Our results regarding the distribution patterns of the Bn*PME*-*GFP* fusion protein show, for the first time in *Brassica napus*, that the PME protein encoded by the Bn*PME* gene sequence used in this work is directed to the cell walls where it may exert its enzymatic activity over pectins *in muro*.

### High pectin esterification and low Bn*PME* expression are associated with cell totipotency and proliferation in the embryogenic pathway

The vacuolated microspore is a very active cell with the capacity to either form the pollen grain via the gametophytic pathway or to be reprogrammed by stress and become a totipotent cell, proliferate and form proembryos and embryos via the embryogenic pathway [23, 47, 63]. Both, vacuolated microspores and early proembryo cells, exhibited low levels of Bn*PME* expression, as well as high levels of esterified pectins, detected by immuno-dot-blot and immunofluorescence assays with JIM7 antibodies.

Recently, some reports have indicated that changes in cell wall mechanics controlled by the esterification status of pectins through PME activity underlie organogenesis initiation and phyllotaxis in *Arabidopsis* [59, 64]. Moreover, biochemical changes in the cell wall that promote its loosening have been reported at early embryo stages, cell wall remodeling that seems to be crucial for proper embryo growth and embryogenesis progression [56, 57]. Therefore, the low levels of Bn*PME* expression that we found at early embryo stages could be key for cell wall loosening to facilitate the necessary growth spurt that is characteristic of this stage.

Previous studies by our group have reported changes in pectin esterification in microspores and microspore-derived embryos in various plant species such as *Capsicum annuum*, *Quercus suber*, *Citrus clementina* and *Olea europaea*, as well as in meristematic and differentiating cells of *Allium cepa* root tips [16, 26, 27, 29, 30, 37, 65]. The comparative analysis performed in the present study, between the in vivo pollen development and the in vitro microspore embryogenic pathway, has allowed the identification of a new differential cell wall feature in the microspore reprogramming towards embryogenesis induced by stress. This feature is the high level of pectin esterification present in the cell walls of vacuolated microspores and proembryo cells — therefore associated with cell totipotency and embryogenesis initiation—but not present in cells that follow the gametophytic programme (differentiated pollen). The modifications in cell wall components and pectin residues have been reported as being crucial for initiating cell responses in relation to cell fate and development [66]. In pepper, cork oak and olive, an unusually thick cell wall under the exine was reported in embryogenic microspores and early proembryos [16, 26, 30]. Moreover, specifically in the intine (the inner wall layer under the exine) a differential reactivity by calcofluor white staining (preferential for cellulosic components) was reported in embryogenic microspores and early proembryos of olive, suggesting that the structure, arrangement and/or amount of the cellulosic components were modified after the embryogenic induction [26]. In this sense, the present results indicate that the high proportion of esterified pectins in cell wall may not only be a marker of proliferation but also a marker of the switch of developmental programme and totipotency. In other words, high levels of non-esterified pectins can be considered as a specific marker for the gametophytic pathway while esterified pectins can be considered a marker of totipotency acquisition and early embryogenic development.

During the gametophytic pathway, differentiated pollen showed low Bn*PME* expression, despite the fact that the immuno-dot-blot and immunofluorescence assays with JIM5 antibodies revealed a high content of non-esterified pectins in the cell wall of bicellular and tricellular pollen. Previous studies reported that expression of PME genes is strongly regulated in a tissue-specific manner [67]. In many plants, multiple isoforms of PME encoded by large multigene families have been found, all of which catalyze the same reaction; moreover, some PME isoforms are constitutively expressed whereas others are only expressed in certain tissues/organs and/or in precise developmental stages (review in [34]). In addition, several studies have reported an increase in PME expression levels at later stages of pollen differentiation related to the pollen tube emission during pollen germination in some species [8, 32, 68], as is the case of *PME48* of *Arabidopsis thaliana* [69]; indeed, in some cases, *PME* expression even continued in the pollen tube [8]. In the present study, analysis of the Bn*PME* gene was approached by using the sequence of the papillar cell PME-like gene characterized in *Brassica napus* [40], one of the few PME gene sequences identified in this species, which did not show expression in differentiated pollen. Therefore, it is possible that another PME gene, perhaps a pollen-specific one, is expressed and acting in the pollen differentiation stage in *Brassica napus*.

## Conclusions

In short, the comparison of the findings between the two developmental pathways, gametophytic and embryogenic, revealed that the change of microspore developmental programme involved changes in pectin esterification levels and Bn*PME* expression which may cause the cell wall remodeling during the process. Our results allowed the in situ identification of defined cell wall distribution patterns of pectin esterification associated with cell totipotency, proliferation and differentiation.

Our results, obtained by several complementary methodologies, show that cell differentiation at advanced stages of microspore embryogenesis involves an increase of Bn*PME* expression, also detected in zygotic embryos, as well as high pectin de-esterification. By contrast, totipotency acquisition and entry into proliferation, that both occur at early stages of stress-induced microspore embryogenesis, were associated with low levels of Bn*PME* expression and high levels of esterified pectins in cell walls. Taken together, our results indicate that PME is transcriptionally regulated during microspore embryogenesis induction and progression, correlating with the de-esterification of pectins, and indicating a possible role of PME in the remodeling of the cell wall during the process. PME induction and de-esterification of pectins may contribute to the temporal regulation of the mechanical properties of embryo cell walls, a process associated with embryo cell differentiation. The findings provide new insights into the possible role of pectin esterification modifications, which likely contribute to cell wall remodeling, in microspore totipotency, embryogenesis induction and progression.

## Abbreviations

DIC, Differential interference contrast; FISH, Fluorescence *in situ *hybridization; GFP, Green fluorescence protein; HGA, Homogalacturonan; JIM, John Innes monoclonal; PME, Pectin methylesterase; PMEI, Pectin methylesterase inhibitor
